# Development and Validation of a Bayesian Network for Supporting the Etiological Diagnosis of Uveitis

**DOI:** 10.3390/jcm10153398

**Published:** 2021-07-30

**Authors:** Yvan Jamilloux, Nicolas Romain-Scelle, Muriel Rabilloud, Coralie Morel, Laurent Kodjikian, Delphine Maucort-Boulch, Philip Bielefeld, Pascal Sève

**Affiliations:** 1 Department of Internal Medicine, Hôpital de la Croix-Rousse, Hospices Civils de Lyon, Université Claude Bernard-Lyon 1, F-69004 Lyon, France; yvan.jamilloux@chu-lyon.fr; 2 Service de Biostatistique et Bioinformatique, Pôle Santé Publique, Hospices Civils de Lyon, Université de Lyon, F-69000 Lyon, France; nicolas.romain-scelle@chu-lyon.fr (N.R.-S.); muriel.rabilloud@chu-lyon.fr (M.R.); coralie.morel@chu-lyon.fr (C.M.); delphine.maucort-boulch@chu-lyon.fr (D.M.-B.); 3 Laboratoire de Biométrie et Biologie Évolutive, Équipe Biostatistique-Santé, CNRS, UMR 5558, F-69100 Villeurbanne, France; 4 Department of Ophthalmology, Hôpital de la Croix-Rousse, Hospices Civils de Lyon, Université Claude Bernard-Lyon 1, F-69004 Lyon, France; laurent.kodjikian@chu-lyon.fr; 5 Department of Internal Medicine, Dijon Bourgogne University Hospital, F-21000 Dijon, France; philip.bielefeld@chu-dijon.fr; 6 Research on Healthcare Performance (RESHAPE), INSERM U1290, Université Claude Bernard Lyon 1, F-69000 Lyon, France

**Keywords:** uveitis, diagnosis, algorithm, artificial intelligence, Bayesian network

## Abstract

The etiological diagnosis of uveitis is complex. We aimed to implement and validate a Bayesian belief network algorithm for the differential diagnosis of the most relevant causes of uveitis. The training dataset (*n* = 897) and the test dataset (*n* = 154) were composed of all incident cases of uveitis admitted to two internal medicine departments, in two independent French centers (Lyon, 2003–2016 and Dijon, 2015–2017). The etiologies of uveitis were classified into eight groups. The algorithm was based on simple epidemiological characteristics (age, gender, and ethnicity) and anatomoclinical features of uveitis. The cross-validated estimate obtained in the training dataset concluded that the etiology of uveitis determined by the experts corresponded to one of the two most probable diagnoses in at least 77% of the cases. In the test dataset, this probability reached at least 83%. For the training and test datasets, when the most likely diagnosis was considered, the highest sensitivity was obtained for spondyloarthritis and HLA-B27-related uveitis (76% and 63%, respectively). The respective specificities were 93% and 54%. This algorithm could help junior and general ophthalmologists in the differential diagnosis of uveitis. It could guide the diagnostic work-up and help in the selection of further diagnostic investigations.

## 1. Introduction

Uveitis is an inflammation of the iris, ciliary body, vitreous, retina or choroid. According to studies, its incidence ranges between 10.5 and 52/100,000 person-years and its prevalence is between 38 and 284/100,000 persons [1,2,3]. In developed countries, it is the fifth most common cause of blindness among the working-age population, mainly because of macular oedema, ocular hypertonia, or retinal ischemia [4]. About 80 causes of uveitis have been described and these can be classified into four main groups. In Western countries, approximately one-quarter of cases are linked to ophthalmologic diseases, one-quarter to systemic diseases fulfilling consensual diagnostic criteria, one-quarter to presumed systemic diseases, and one-quarter have an unexplained origin [5]. Uveitis of unexplained origin, also known as idiopathic uveitis, represents 23–44% of cases according to recent studies [6,7,8,9,10,11].

Diagnosis of the different conditions causing uveitis is based on the presence of different features, such as genetic (e.g., HLA-B27, HLA-A29) and ethnic factors, epidemiologic characteristics (i.e., age, gender, ancestry), anatomical localization (i.e., anterior, intermediate, posterior, or panuveitis), the laterality (i.e., uni-or bilateral), and the chronicity (i.e., acute, recurrent, or chronic) [12,13]. Other ophthalmologic characteristics may also orient the diagnosis, such as the granulomatous character, the existence of ocular hypertonia, synechia, or retinal vasculitis (venous and/or arterial, occlusive), and the occurrence of single or multiple retinochoroidal lesions [6,13]. The differential diagnosis of uveitis is often complex for junior, general ophthalmologists or physicians. However, a prompt diagnosis may lead to better management of the patient, especially if they have systemic involvement. Several algorithms for etiological diagnosis have been proposed [14,15,16,17,18]. For complex cases, a collaborative network of ophthalmologists and internists/rheumatologists has shown better efficiency [19,20,21]. A systematic approach to the etiological diagnosis, although not inferior to a free strategy, is less time consuming and overall less costly [22].

Bayes’ theorem links the degree of belief in a proposition before and after accounting for new information. This theorem has been used as a basis for the development of some artificial intelligence algorithm, named Bayesian belief networks. The use of such networks has been previously reported for the differential diagnosis of a wide variety of medical conditions, such as dementia or breast lesions [23,24]. Gonzalez-Lopez et al. applied this technique to the differential diagnosis of anterior uveitis [25].

Using two independent datasets of uveitis patients, the aim of this study was to implement and validate a Bayesian belief network algorithm for the differential diagnosis of the most relevant causes of uveitis, based on epidemiological and anatomoclinical characteristics.

## 2. Materials and Methods

### 2.1. Datasets

The training dataset was composed of all incident cases of uveitis admitted to the department of internal medicine at the Croix-Rousse University Hospital, Lyon, between 1 January 2003 and December 2016. The test dataset was composed of all incident cases of uveitis admitted to the department of internal medicine at the Dijon Bourgogne University Hospital, Dijon, between 1 January 2015 and December 2017. For all patients, the uveitis diagnosis was achieved after an ophthalmological examination. Depending on the anatomical classification [13,26], all uveitis patients underwent a standardized screening protocol, which has been reported previously [19]. Patients with an etiological diagnosis in progress were not included. Patients with uveitis related to pure ophthalmological entities, diagnosed solely by ophthalmological examination, and referred for treatment or for ruling out differential diagnoses, as well as uveitis occurring during a previously diagnosed disease, and patients with missing data in studied variables were excluded.

### 2.2. Definitions

The Standardization of Uveitis Nomenclature (SUN) was used throughout this study for the anatomic classification of uveitis [26]. Infectious uveitis was diagnosed by appropriate microbiological tests (culture or polymerase chain reaction in ocular fluid, blood, cerebrospinal fluid or tissue biopsy, serology or therapeutic test for fastidious bacteria). Intraocular tuberculosis was diagnosed using Gupta’s criteria [27]. We used the ASAS criteria for spondyloarthritis [28], the international study group criteria for Behcet’s disease [29], the revised diagnostic criteria for Vogt-Koyanagi-Harada disease [30], the international conference consensus criteria for the diagnosis of birdshot chorioretinopathy [31], and the 2010-revised McDonald’s criteria for multiple sclerosis [32]. Most of these criteria (e.g., ASAS criteria) are not intended for diagnostic purposes but are classification criteria used to include patients in clinical trials or studies. For sarcoidosis, we used the international criteria for the diagnosis of sarcoidosis [17,33]. In the absence of histological proof, we used Abad’s modified criteria [34]; patients had presumed sarcoid uveitis if they had at least two of the following four criteria: typical changes on chest X-ray or CT scan, a predominantly CD4 lymphocytosis on BAL fluid analysis, an elevated ACE or an 18-fluorodeoxyglucose (18-FDG) uptake on scintigraphy. They had indeterminate sarcoid uveitis when only one criterion was met.

### 2.3. Study Factors

We selected three simple and relevant demographic factors: (i) age at uveitis onset, divided into four groups (<40 years, 40–50 years, 50–60 years and >60 years) to include this factor as a qualitative variable; (ii) ethnicity, divided into Caucasians, North Africans, sub-Saharan Africans, Asians, and others; and (iii) gender. These variables were chosen on the basis of various previous epidemiological studies on the causes of uveitis (1–12). Anatomo-clinical characteristics of uveitis included: anatomical type of uveitis and chronicity [26], laterality, granulomatous character, ocular hypertension and vasculitis. Five types of uveitis were considered: anterior, intermediate, posterior uveitis, panuveitis, and combined uveitis. Granulomatous uveitis was defined as the presence of large keratic precipitates or iris nodules. Ocular hypertension was defined as a chronically elevated intraocular pressure (>21 mmHg).

### 2.4. Selected Uveitis Causes

**Figure 1:**
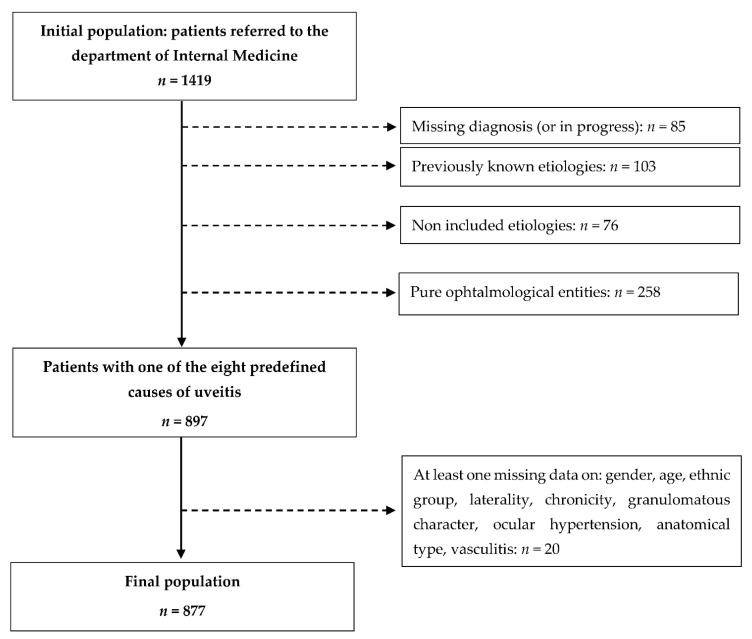
Flow chart for the training dataset.

Eight causes of uveitis were selected for the study: spondyloarthritis and HLA-B27-related uveitis, Behçet’s disease, sarcoidosis, multiple sclerosis, tuberculosis, lymphoma, idiopathic uveitis, and one category grouping a small number of other inflammatory diseases, considered as a unique etiology. In the training dataset, the etiology of uveitis corresponded to one of the selected causes for 897 patients and, of these, 877 had no missing data on the factors studied (Figure 1). The number of patients included in the test dataset was 154.

### 2.5. Bayesian Networks

**Figure 2:**
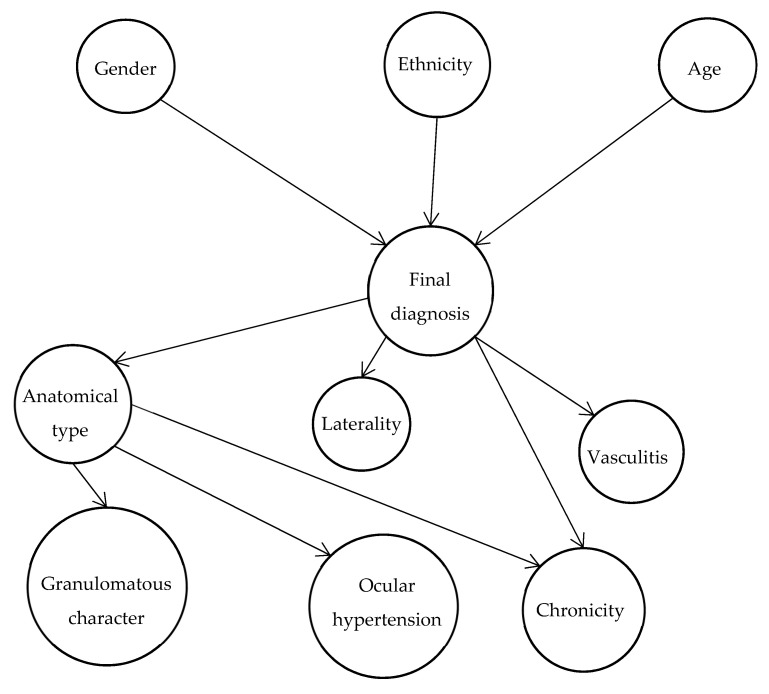
Structure of the Bayesian network for etiologic diagnosis of uveitis.

Bayesian belief networks (BN) are probabilistic graphic models. They are used to represent uncertain knowledge, to calculate conditional probabilities and can be used for decision support systems. Their graphical representation allows for an intuitive interpretation of the causality links among studied variables, while retaining the mathematical property necessary to compute estimates of their parameters [35]. The structure of the network was built with the help of an experienced clinician (Figure 2).

In this network, the central node is the etiology of uveitis, which depends on age, gender and ethnicity, and the characteristics of uveitis depend on the etiology of the disease. The purpose is to estimate the Bayesian network parameters from the given network structure and the complete data. Due to the absence of all possible permutations in the training dataset (i.e., not all configurations of sociodemographic and clinical parameters exist among the patients), maximum likelihood estimation was not feasible. We resorted to use a Bayesian approach to estimate the parameters of each node. All variables being categorical, a Dirichlet distribution corresponding to a uniform distribution was used as prior distribution with a weight corresponding to an additional number of patients of 2. This approach allows an estimation of the posterior joint probabilities of the nodes knowing their parents, given the training dataset. Network training was carried out using all complete cases of the training dataset, in which all patients had an etiological diagnosis established by expert physicians. To assess the algorithm performance, internal and external validations were carried out. Internal validation was conducted using a Monte Carlo cross validation (MCCV) method. At each iteration, the training dataset was split into two groups. The parameters were estimated on the first group comprising 80% of the patients selected at random, with stratification on each etiology to ensure inclusion of all etiologies and validated on the group of remaining patients. The performance of the network was quantified by the precision corresponding to the proportion of correctly classified patients, the sensitivity and the specificity for each etiology, using the mean of the estimates obtained on 5000 iterations. These performance indicators were estimated for the most probable diagnosis and the two most probable diagnoses. Confidence intervals (CI) were built using the variance combining the mean of the variances estimated at each iteration and the variance between iterations [36]. Precision, sensitivity, and specificity were also estimated on the test dataset used for external validation. All the statistical analysis and the development of the Bayesian network was performed using the R software, version 3.6.1 (R Foundation for Statistical Computing, Vienna, Austria), and the packages bnlearn and gRain (with their respective dependencies) [37,38].

### 2.6. Ethic Statement

According to French law (no. 2004-806, 9 August 2004), and because the data were collected retrospectively and patient management was not modified, this study did not require research ethics committee approval. However, the cohort study received approval from the local ethics committee (Hospices Civils de Lyon) in February 2019 (No. 19–31) and was registered on clinicaltrials.gov (NCT 03863782).

## 3. Results

### 3.1. Description of the Study Populations

#### 3.1.1. Training Dataset

In this population, anterior uveitis represented the most frequent anatomic type (35.5%), followed by panuveitis (30.4%, Table 1). Uveitis was chronic in about 67% of the patients and bilateral in about 59%. It was idiopathic in 49% of the patients, and sarcoidosis represented the first identified etiology (22%) followed by the group of spondyloarthritis and HLA-B27-related uveitis (13.6%).

#### 3.1.2. Test Dataset

In the population used for external validation, the proportion of anterior uveitis was twice as high as in the training population (67%). Uveitis was unilateral in 74% of the cases and idiopathic in 67%. As in the training population, spondyloarthritis and HLA-B27-related uveitis, and sarcoidosis were the two most frequently identified etiologies (Table 1).

### 3.2. Precision, Sensitivity and Specificity Estimates

The cross-validated estimate of the Bayesian network precision was 54% (95% CI: 45–64%) when the most probable diagnosis was considered and reached 80% (95% CI: 77–84%) when the two most probable diagnoses were considered. It was similar in the test dataset with an estimate at 49% (95% CI: 39–59%) when the most probable diagnosis was considered and 85% (95% CI: 83–87%) when the two most probable diagnoses were considered.

When the most probable diagnosis was considered, the highest sensitivity was obtained for spondyloarthritis and HLA-B27-related uveitis with a cross-validated estimate of 76% (95% CI: 71–81%), followed by the idiopathic uveitis with a cross-validated estimate of 70% (95% CI: 63–76%) (Table 2). The specificity was estimated respectively at 93% (95% CI: 93–94%) and 55% (95% CI: 45–65%). When the two most probable diagnoses were considered, the cross-validated estimate of sensitivity reached 92% (95% CI: 91–93%) for spondyloarthritis and HLA-B27-related uveitis and 96% (95% CI: 95–96%) for idiopathic uveitis, at the cost of a decrease of the specificity, which was estimated respectively at 85% and 3%.

In the test dataset, the number of patients was very low for most of etiologies except for spondyloarthritis and HLA-B27-related uveitis and idiopathic uveitis. For etiologies with few patients, estimates should be interpreted with caution. When the most probable diagnosis was considered, the sensitivity was estimated at 63% (95% CI: 55–72%) for spondyloarthritis and HLA-B27-related uveitis and 52% (95% CI: 42–62%) for idiopathic uveitis (Table 3). The specificity was estimated respectively at 54% (95% CI: 44–64%) and 65% (95% CI: 57–73%). When the two most probable diagnoses were considered, the sensitivity estimate increased to 83% and 99% respectively, at the cost of a decrease of the specificity estimate to 33% and 4% respectively.

## 4. Discussion

This Bayesian belief network allowed the identification of uveitis etiology with good performance when the two most likely diagnoses were sought. The estimate obtained by cross-validation in the training dataset concluded that the uveitis etiology determined by experts matched one of the two most likely diagnoses in at least 77% of the cases. Using the estimate obtained in the test dataset, this probability reached at least 83%.

Bayesian belief networks have been previously reported for the differential diagnosis of several conditions [23,24], including anterior uveitis [25]. Our results are similar to those obtained with the Bayesian network developed by Gonzalez-Lopez et al. [25]. They found that the etiology determined by the senior clinician matched the first or the second most probable diagnosis given by the algorithm in about 80% of the cases. Yet, these authors only focused on anterior uveitis and did not include the age parameter, nor the category of idiopathic uveitis, which is the predominant etiology in our cohort. As in their study, the specificities determined by our Bayesian belief network were high, allowing the exclusion of less likely etiologies. Sensitivities, on the other hand, are highly variable; diseases for which diagnostic criteria exist are more often correctly identified than diseases for which diagnosis is one of exclusion. A limitation of the study is the relatively small sample sizes for several etiologies in the training dataset and even more so in the test dataset that did not allow for reliable estimates of the sensitivity and specificity of the Bayesian network for these etiologies.

However, this study continues the current development of artificial intelligence in diagnostic procedures, such as uveitis [18,39]. Indeed, many efforts are being made to assist the clinician with automated computer-based systems, usually termed diagnostic decision support systems (DDSS) [40,41]. Other tools, which are not based on Bayesian belief networks, exist for the etiological diagnosis of uveitis. One app, named Uveitis Doctor (Lara-Medina, Alcazar de San Juan, Spain) is currently available on iOS (https://apps.apple.com/es/app/uveitis-doctor; accessed on 30 July 2021), comprises more than 50 uveitis syndromes and the inference engine is based on decision trees. Another DDSS, named Uvemaster, contains a knowledge base of 88 uveitis syndromes, each comprising 76 clinical items [39] and was shown to have a 73.9% sensitivity. These apps may improve the clinical management of uveitis by offering potential diagnoses and reducing the number of cases labeled as idiopathic uveitis. However, the knowledge base includes diagnoses with very high specificity tests and ophthalmological entities that can be easily recognized by the ophthalmologist.

For the time being, diagnostic algorithms are mainly efficient for the most common diagnoses. However, the major problem faced by specialists is rather to determine rare or difficult diagnoses, in order not to leave too many patients in diagnostic uncertainty or with a diagnosis of “undetermined origin”. Thus, algorithms that are more complex remain to be developed. These algorithms would include a greater number of parameters, with a higher level of accuracy and complexity, including extra-ophthalmological features. It will therefore be necessary to determine the most specific and relevant parameters. On the other hand, the use of easily accessible and simple to collect parameters, such as those used in our study, are a very good starting point in the diagnostic process. We designed this algorithm as an aid for (junior) inexperienced clinicians. This algorithm is not to be seen as an exact test, but rather as a useful tool to help in medical decision-making. It can highlight candidate etiologies and help in the selection of further diagnostic investigations. A prospective study will be required to test the diagnostic performance of the algorithm.

Our study has several limitations, the first one being its retrospective nature, usually leading to a large amount of missing data. However, our tertiary centers have set up a systematic approach to the etiological diagnosis of uveitis and, actually, only 20 of the 1051 patients did not have a complete set of data. Another limitation is linked to the recruitment of patients since our tertiary center is specialized in the management of uveitis and in extrapulmonary sarcoidosis. This may have led to the inclusion of more severe cases. In addition, the country where the study was performed is not in an endemic area, therefore the results cannot be extrapolated to such regions. We used ethnicity criteria based on the country of birth. However, the relevance of these criteria, now and in the future, needs to be questioned, as it does not consider the heterogeneity within these geographical groups and the effects of environment. Current and future population movements may also lead to changes in the epidemiology of uveitis. As the study was performed in France, the Caucasian group actually represents 77% of the individuals in the data set on which the model was developed. The other ethnic groups are very much in the minority. As a result, the information provided by the ethnic group to predict the etiology is weak and this represents a limitation of the study. We did not perform a sensitivity analysis to assess the weight of the various demographic factors, because of small sample subgroups. Lastly, some conditions like herpetic uveitis, syphilis or others have not been included among the etiologic candidates.

The over-representation of idiopathic uveitis (i.e., of undetermined origin) is linked to the increasing medical knowledge and better recognition and classification of these entities. As these advances are continuous, algorithms must be prospectively modified. Two recent studies have respectively shown that an etiology can finally be found in 18% and 29% of cases of uveitis initially classified as idiopathic [42,43]. Algorithms should therefore be dynamic and adaptive.

Importantly, the main criteria for guiding the diagnostic approach, and thus the algorithm, are the characteristics of the uveitis [26]. A careful and repeated ophthalmological examination is therefore required to allow an accurate description of uveitis anatomy and evolution. The ophthalmologist has a crucial role, upstream of the Bayesian network (pre-test), to ensure maximum performance.

## 5. Conclusions

Our study represents a first step confirming the interest and feasibility of diagnostic algorithms in the workup of uveitis. It provides leads for future developments but needs to be confirmed by prospective and larger studies.

## Figures and Tables

**Table 1:** Description of the characteristics of the patients in training dataset and in the test dataset.

		Training Dataset 877 (100%)	Test Dataset 154 (100%)
Age at diagnosis (years)	<40	315 (35.9%)	74 (48.1%)
	40–50	146 (16.6%)	24 (15.6%)
	50–60	134 (15.3%)	19 (12.3%)
	>60	282 (32.2%)	37 (24.0%)
Sex	Female	534 (60.9%)	78 (50.6%)
	Male	343 (39.1%)	76 (49.4%)
Ethnic group	Caucasian	676 (77.1%)	127 (82.5%)
	Sub-Saharan African	12 (1.4%)	5 (3.2%)
	North African	157 (17.9%)	22 (14.3%)
	South American	11 (1.3%)	0
	Other	7 (0.8%)	0
	Asian	14 (1.6%)	0
Anatomic type	Anterior uveitis	311 (35.5%)	103 (66.9%)
	Combined uveitis	104 (11.9%)	6 (3.9%)
	Panuveitis	267 (30.4%)	19 (12.3%)
	Posterior uveitis	111 (12.7%)	14 (9.1%)
	Intermediate uveitis	84 (9.6%)	12 (7.8%)
Chronicity	Acute	292 (33.3%)	144 (93.5%)
	Chronic	585 (66.7%)	10 (6.5%)
Laterality	Bilateral	515 (58.7%)	40 (26.0%)
	Unilateral	362 (41.3%)	114 (74.0%)
	Not available	3 (0.3%)	0
Granulomatous character	No	614 (70.0%)	120 (77.9%)
	Yes	263 (30.0%)	34 (22.1%)
	Not available	8 (0.9%)	0
Vasculitis	No	793 (88.4%)	132 (85.2%)
	Yes	104 (11.6%)	23 (14.8%)
Ocular hypertension	No	804 (91.7%)	137 (89.0%)
	Yes	73 (8.3%)	17 (11.0%)
	Not available	9 (1.0%)	0
Etiology	Idiopathic uveitis	431 (49.1%)	103 (66.9%)
	Sarcoidosis	191 (21.8%)	8 (5.2%)
	Spondyloarthritis & HLA-B27-related	119 (13.6%)	30 (19.5%)
	Tuberculosis	59 (6.7%)	1 (0.6%)
	Behcet’s disease	30 (3.4%)	3 (1.9%)
	Multiple sclerosis	22 (2.5%)	2 (1.3%)
	Lymphoma	15 (1.7%)	2 (1.3%)
	Other inflammatory diseases	10 (1.1%)	5 (3.2%)

**Table 2:** Sensitivity and specificity of the Bayesian network to identify the uveitis etiology using the most probable diagnosis and the two most probable diagnoses: estimates based on the Monte Carlo cross-validation carried out in the training dataset.

Etiology	Most Probable Diagnosis		Two Most Probable Diagnoses	
	Sensitivity (95% CI)	Specificity (95% CI)	Sensitivity (95% CI)	Specificity (95% CI)
Idiopathic	0.70 (0.63; 0.76)	0.55 (0.45; 0.65)	0.96 (0.95; 0.96)	0.03 (0.03; 0.03)
Sarcoidosis	0.33 (0.26; 0.41)	0.90 (0.89; 0.91)	0.69 (0.62; 0.76)	0.52 (0.42; 0.62)
Spondyloarthritis & HLA-B27-related	0.76 (0.71; 0.81)	0.93 (0.93; 0.94)	0.92 (0.91; 0.93)	0.85 (0.83; 0.87)
Tuberculosis	0 (0; 0)	0.97 (0.97; 0.97)	0.23 (0.19; 0.28)	0.92 (0.92; 0.93)
Behcet’s disease	0.67 (0.59; 0.74)	0.95 (0.95; 0.95)	0.67 (0.59; 0.74)	0.94 (0.93; 0.94)
Multiple sclerosis	0.20 (0.17; 0.24)	0.99 (0.99; 0.99)	0.40 (0.31; 0.49)	0.97 (0.97; 0.97)
Lymphoma	0 (0; 0)	1 (1; 1)	0.67 (0.59; 0.74)	0.97 (0.97; 0.97)
Other inflammatory etiologies	0 (0; 0)	0.99 (0.99; 0.99)	0 (0; 0)	0.99 (0.99; 0.99)

**Table 3:** Sensitivity and specificity of the Bayesian network to identify the uveitis etiology using the most probable diagnosis and the two most probable diagnoses: estimates in the test dataset.

Etiology	Most Probable Diagnosis		Two Most Probable Diagnoses	
	Sensitivity (95% CI)	Specificity (95% CI)	Sensitivity (95% CI)	Specificity (95% CI)
Idiopathic	0.52 (0.42; 0.62)	0.65 (0.57; 0.73)	0.99 (0.99; 0.99)	0.04 (0.04; 0.04)
Sarcoidosis	0.38 (0.29; 0.46)	0.98 (0.98; 0.98)	0.50 (0.4; 0.6)	0.72 (0.66; 0.78)
Spondyloarthritis & HLA-B27-related	0.63 (0.55; 0.72)	0.54 (0.44; 0.64)	0.83 (0.81; 0.86)	0.33 (0.25; 0.40)
Tuberculosis	0 (0; 0)	1 (1; 1)	0 (0; 0)	0.97 (0.97; 0.98)
Behcet’s disease	0 (0; 0)	1 (1; 1)	0 (0; 0)	1 (1; 1)
Multiple sclerosis	0 (0; 0)	1 (1; 1)	0 (0; 0)	1 (1; 1)
Lymphoma	0 (0; 0)	1 (1; 1)	0 (0; 0)	1 (1; 1)
Other inflammatory etiologies	0 (0; 0)	1 (1; 1)	0 (0; 0)	1 (1; 1)

## Data Availability

The data presented in this study are available on reasonable request from the corresponding author.
